# Imbalance and breakout in the post-epidemic era: Research into the spatial patterns of freight demand network in six provinces of central China

**DOI:** 10.1371/journal.pone.0250375

**Published:** 2021-04-22

**Authors:** Yin Huang, Runda Liu, Shumin Huang, Gege Yang, Xiaofan Zhang, Yin Qin, Lisha Mao, Sishi Sheng, Biao Huang

**Affiliations:** 1 School of Logistics and Transportation, Central South University of Forestry and Technology, Changsha, Hunan, China; 2 Key Laboratory of Traffic Safety on Track (Central South University), Ministry of Education, Changsha,China; 3 Joint International Research Laboratory of Key Technology for Rail Traffic Safety, Changsha, China; 4 National & Local Joint Engineering Research Center of Safety Technology for Rail Vehicle, Changsha, China; Institute for Advanced Sustainability Studies, GERMANY

## Abstract

This study aims to explore the freight demand network spatial patterns in six provinces of central China from the perspective of the spread of the epidemic and the freight imbalance and breakout. To achieve this purpose, the big data of “cart search” demand information provided by small and medium freight enterprises on the freight information platform are analyzed. 343,690 pieces of freight demand big data on the freight information platform and Python, ArcGIS, UCINET, and Gephi software are used. The results show that: (1) The choke-point of unbalanced freight demand network is Wuhan, and the secondary choke-points are Hefei and Zhengzhou. (2) In southern China, a chain reaction circle of freight imbalance is formed with Wuhan, Hefei, and Nanchang as the centers. In northern China, a chain reaction circle of freight imbalance is formed with Zhengzhou and Taiyuan as the centers. (3) The freight demand of the six provinces in central China exhibits typical characteristics of long tail distribution with large span and unbalanced distribution. (4) The import and export of freight in different cities vary greatly, and the distribution is unbalanced. This study indicates the imbalance difference, chain reaction, keys and hidden troubles posed by the freight demand network. From the perspectives of freight transfer breakout, freight balance breakout, freight strength breakout, and breakout of freight periphery cities, we propose solutions to breakouts in the freight market in six provinces of central China in the post-epidemic era.

## Introduction

At present, the novel Coronavirus (COVID-19) outbreak has spread to more than 140 countries and regions and ’the global economy is estimated to have contracted 4.3 percent in 2020, representing the deepest recession since World War II. Director-General Tedros of the WHO has stated that the effect of the coronavirus will be felt for decades to come [1]. During this epidemic, China has achieved a phased victory in the battle, however, the severe shock of the epidemic has made China’s economic market more complex and interlaced. The characteristics of non-linearity, chain reactions, and feedback cycles make it difficult to predict how changes will affect the entire economic system. This has also exposed the vulnerability of some markets to the spread and volatility of the epidemic. Unfortunately, our knowledge of the topology is limited, especially on the shape, pattern, and connectivity that will determine how a supply shock affects global production under the COVID-19 epidemic. The real threat is to expose previously unknown choke-points in the economic web. Many market networks are, *per se*, hierarchical and unbalanced, with the network’s key central point having an extraordinary influence. These centers are likely to become the choke-points that lead to market imbalances and even shocks, and when they are cut off, economic activity will be severely disrupted [2].

Some studies focus on the COVID-19 battle. Population migration and urban traffic are two important aspects of these studies [3–5], and the data analysis in geographical flow is a useful tool for the spread of the COVID-19 epidemic analysis. These techniques were adopted to forecast the levels of an epidemic [5], but few studies predicted and analyzed the market economy in the post-epidemic era. Xu *et al* pointed out that the foundation of the market economy lies in the full free flow of elements and resources, including people, materials, and information [3]. Hence, logistics, especially for the materials transportation, that is freight flow, is also an important element of the market economy. Our study focuses on the freight flow and freight economic market development in the post-epidemic era based on the freight demand big data analysis during the spread of COVID-19.

After experiencing the COVID-19 shock, the survival and development of small and medium enterprises are more difficult. In the post-epidemic era, it is imperative to find opportunities from the new crisis and seek new development opportunities. Based on the above problems, we crawled 343,690 pieces of cargo demand data through the Haoyun Logistics Website (http://www.haoyun56.com), to ascertain the evolution characteristics of topological structure of freight demand network in six provinces of central China from the perspective of the spread of the epidemic, we then assessed structural changes of different center nodes in the six provinces of central China to clarify the six provinces of central China in terms of freight demand market imbalance and even the key node of the shock from the perspective of the spread of the epidemic. Afterwards, the coping mechanism for small and medium freight enterprises in six provinces of central China in the post-epidemic era is revealed to find opportunity from the new crisis centering on “imbalance” and “breakout”. Based on the analysis of the spatial patterns of freight demand in the six provinces of central China from the perspective of the epidemic spread, we analyze the imbalance of the freight demand market in these regions, and explore methods and coping strategies for small and medium freight enterprise development in the post-epidemic era from the perspective of urban freight flow analysis during the spread of COVID-19.

The urban logistics system is a support network system to meet the needs of cities. Due to the increasingly expanding competitive environment, it shows the characteristics of dispersed resources and low efficiency. Imbalance between supply and demand is an important issue in logistics supply and demand network. It is also an important factor that causes many social and economic problems [6]. The urban freight market is no exception, and the phenomenon of “high consumption and low energy” in small and medium freight enterprise markets is becoming more serious. With the rapid growth of road freight volumes, the problems in the freight market are becoming more prominent [7, 8]. 70% of China’s freight enterprises are small and medium enterprises, and they have been seeking a breakthrough in the dilemma posed by the imbalance between supply and demand. Affected by the spread of the epidemic, the supply and demand of small and medium freight enterprises are becoming more imbalanced. They have been living in the dilemma and gap between supply and demand for years and need an urgent breakthrough in this turmoil.

Many scholars have researched the structural characteristics of freight networks and have achieved a series of excellent results. These results describe the structural characteristics and evolution trends of different freight markets from different aspects. Yamada *et al*. established a network equilibrium model based on supply chain and transportation network to determine the transportation cost in the freight supply chain network [9]. Cao and Li developed a transport efficiency analysis framework based on spatial regional units, which provided a scientific reference for optimizing the spatial layout of regional transport infrastructure [10]. Li and Ma extracted microscopic freight data directly based on express way-bills, and discussed the spatial patterns, structural features, and functional organization of China’s express transport network [11]. Mi used 55623 O-D data from water freight, road freight, railway freight, and air freight on the freight website in 2015. Based on the perspective of freight flow, he performed regression analysis to identify the current status of logistics links and their influencing factors in the Yangtze River Economic Belt [12]. However, with the continuous development of the freight market in recent years, its network features of networking and complexity are becoming increasingly obvious. Its complex structural characteristics, dynamic formation process, and overly complicated representation have become major problems in the structural characteristics in logistics network analysis [13, 14]. Its topological properties require urgent attention [2, 15]. Particularly during the novel Coronavirus pandemic, 12 prefecture-level cities and one autonomous prefecture in Hubei Province, as comprehensive transportation hubs in six provinces of central China, adopted the measure of “closed city management”. The effective absence of some important freight hubs such as Wuhan led to the imbalance between supply and demand in the freight market of the six provinces of central China, and the vulnerability of their demand network became prominent. Dubie *et al*. found that for the spatial patterns of various logistics activities, a more multi-dimensional and broader perspective is needed [16]. The cyberspace-based structure of freight market demand under the influences of major public health events is one of them. It is necessary to study the complexity, dynamics, and multi-module characteristics of the freight demand network in this complex and changeable environment.

## Data and methods

### Date sources

In this study, Python software is used to edit programs and crawl 343,690 pieces of freight data information from February 6 to 18, 2020 on the Haoyun Logistics Website (http://www.haoyun56.com), which is one of the largest Internet freight information platforms in China. The data pertain to “cars looking for goods, goods looking for cars”, that is “car search”, on the website http://www.haoyun56.com, and can represent the freight demand of small and medium freight enterprises between cities. This study is expressed in terms of two parameters, namely freight frequency and freight volume. The more the cities that are connected by freight, the more frequent the freight demand. Meanwhile, a city with more freight indicates a greater demand for freight in that city. To establish the intercity freight demand network, we set the origin, destination, traffic volume, and time (four parameters), and connect the freight information between the starting city and arriving city to form the O-D (origin-destination) dataset of freight demand between cities based on big data information of freight demand in six provinces of central China.

All of the inter-city freight data is from the GitHub database at https://github.com/Runda-Liu/research-into-the-spatial-patterns-of-freight-demand-network-in-six-provinces-of-central-China-data and made available under License CCBY4.0. The Geo map data is from openstreetmap.org and made available under the License CCBY 4.0 whose full text can be found at: https://www.openstreetmap.org/copyright.

### Methods

#### (1) Network analysis

UCINET and Gephi software are used to analyze the intercity freight demand network of six provinces of central China from three aspects, namely the topology, centrality (including degree centrality, weighted degree centrality, closeness centrality, and betweenness centrality) and core-periphery structure, in combination with Python software.

#### (2) Spatial interpolation analysis

Inverse distance weighted (IDW) features in ArcGIS are used for the spatial interpolation analysis of degree centrality in the freight network.

$$Wi=\frac{h_{i}^{-p}}{\sum_{j=1}^{n}h_{j}^{-p}}$$ (1)

*p* = 2, *h*_*i*_ is the distance between the discrete point and the interpolation point, *w*_*i*_ denotes the interpolation weight.

$$\hat{Z}(s0)=\sum_{i=1}^{N}wiZ(si)$$ (2)

*Z*(*Si*) denotes the degree centrality value at position *I*, *w*_*i*_ is the interpolation weight at position *I*, *s*_*0*_ represents the predicted position, and *N* is the practical measurement values of degree centrality.

## The overall spatial patterns of freight demand network in six provinces of central China from the perspective of epidemic spread

### The overall spatial patterns of the freight demand network in six provinces of central China

To obtain the freight demand network connection diagram of six provinces in the central region, Gephi software is employed to calculate the degree centrality of the relevant city nodes based on the freight demand data in the central region (343,690 items in total) crawled from the Haoyun Logistics Website (http://www.haoyun56.com/). It can be found that Wuhan and Hefei have high value in the freight demand network, followed by provincial capitals such as Zhengzhou, Taiyuan, Changsha, and Nanchang. This finding shows that, during the epidemic, the provincial capital city has a dominant position in the freight demand network, and its freight demand is greater, which is related to the concentration of freight enterprises in the central city of the provincial capital. Wuhan, with the highest value, has the highest freight demand during the epidemic period which is attributed to the implementation of a “city closure” measure and the traffic control on the import and export of goods during the epidemic.

### The strength of freight demand network in six provinces in central China

**Fig 1:**
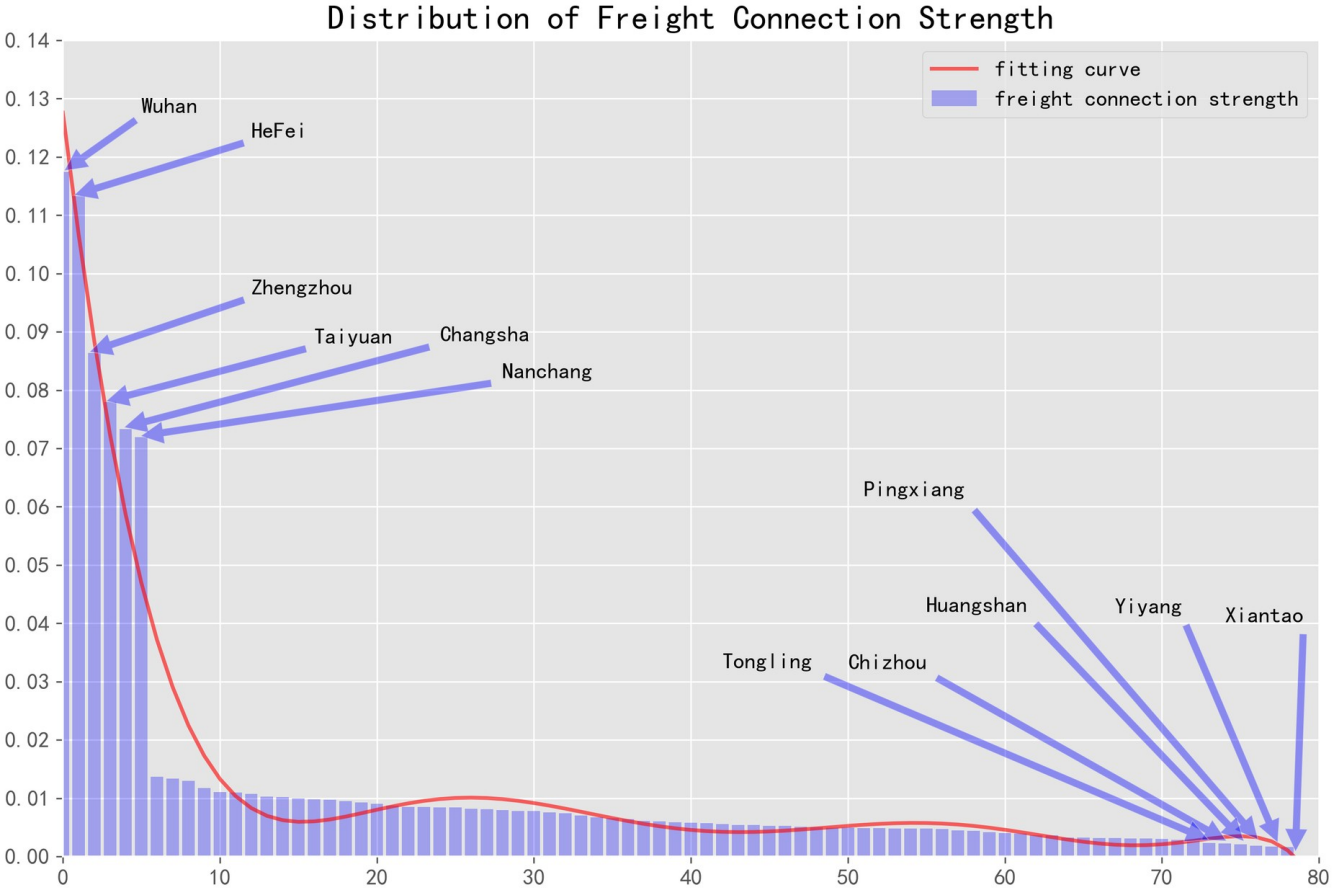
Distribution of the freight demand network strength among six provinces in central China during the spread of the epidemic.

In the theory of complex networks, a scale-free network is a severely heterogeneous network. Most network nodes only have connections or contacts with a few key nodes in the network, such as Wuhan. Relevant statistical variables show that the freight demand network of six provinces in central China follows a power-law distribution across its complex network. By drawing the distribution map of the proportion in freight demand network intensity from February 6 to 18, 2020 (Fig 1), the freight demand of six provinces in central China presents a typical long-tail distribution characteristic. The distribution span and proportion of freight demand intensity between cities are not balanced, indicating that freight demand in six provinces of central China displays a typical uneven distribution during the period of the epidemic. Cities such as Wuhan, Hefei, Zhengzhou, Taiyuan, Changsha, and Nanchang have higher demand; cities such as Tongling, Chizhou, Huangshan, Pingxiang, Yiyang, and Xiantao have lower demand (Fig 1).

We use SPSS software to fit the power function of the distribution chart of demand intensity of urban freight network in six provinces of central China (as shown in Table 1). The results show that *R*^2^ is greater than 0.8 and Sig is less than 0.001, which fits the power function. This indicates that the freight demand network of six provinces in central China conforms to the characteristics of a scale-free network and the related Pareto law. It shows that the frequency of freight transport in 18 cities (about 21% of 83 cities in six provinces of central China), such as Wuhan, Hefei, Zhengzhou, Taiyuan, and Changsha, accounts for more than 70% of the freight frequency.

**Table 1:** Fitting the distribution of inter-city freight demand network strength in six provinces of central China during the epidemic period.

Power function equation	*R*^2^	*F*	*S*ig
0.306*x^−1.109^	0.803	335.055	0.000

### Network analysis of freight demand impact in six provinces of central China

**Fig 2:**
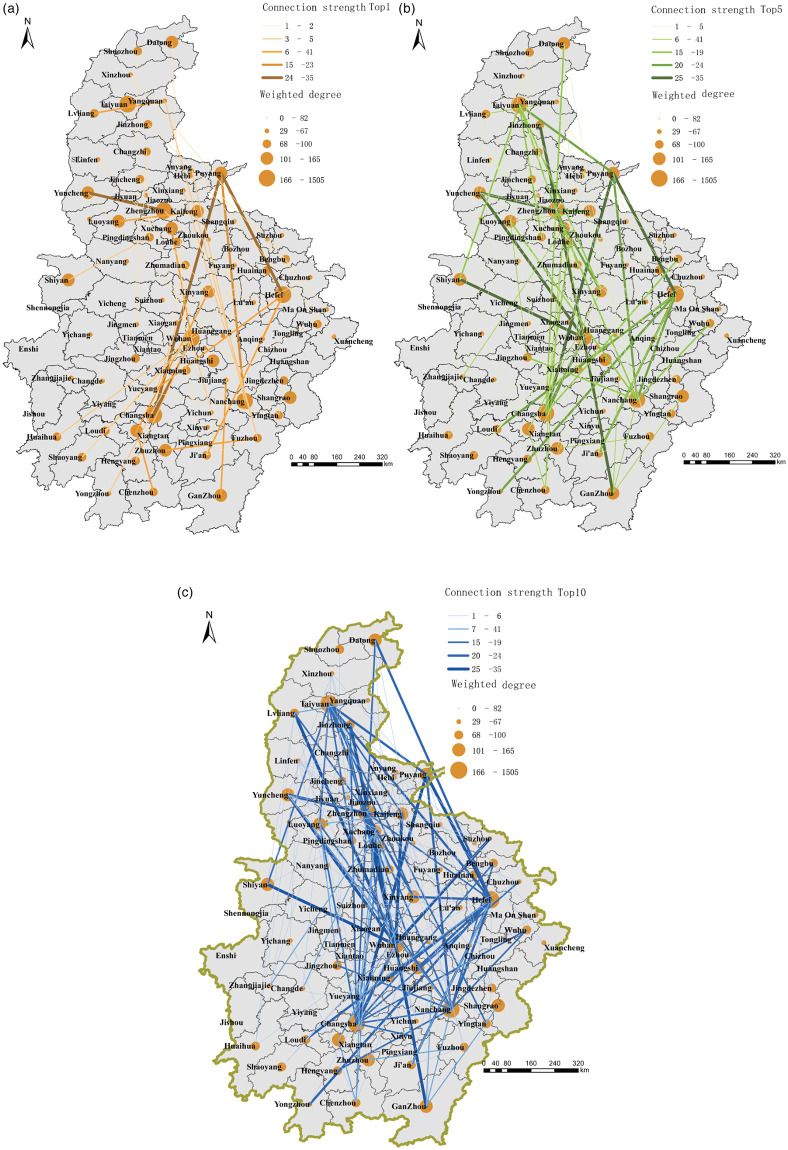
Connection patterns of top 1, 5, and 10 cities in terms of the freight demand impact network in six provinces of central China. a. The first contact map of freight demand. b. The contact map of the top 5 freight demand cities. c. The contact map of the top 10 freight demand cities.

Using ArcGIS 10.6 software platform, the O-D matrix is constructed through a network analyst module, and the O-D network connection path is thus visualized. The freight frequency in O-D data is used as the freight connection strength between the two cities to assess the connection strength between the six cities in central China. Our study selects the top 1, top 5, and top 10 cities with the strength of each city to analyze city networks. By strengthening the network interweaving, the network system characteristics of six provinces in central China are ascertained, and the top 1, 5, and 10 networks of freight links among cities are plotted to analyze the pattern of freight connection network in six provinces of central China (Fig 2).

The overall network density is relatively low (at 0.081) and is affected by the overall freight demands in six provinces of central China. This is because many cities in six central provinces have not yet opened freight stations, and the inter-city freight connections in six provinces are weakly connected due to the closure of cities and roads during the epidemic. The freight demand impact network of six provinces in central China presents a “multi-center and radiation” pattern (Fig 2), with Wuhan, Hefei, Nanchang, Zhengzhou, Taiyuan, and Changsha as the center, affecting cities in a radial pattern. It shows that these seven cities are the central nodes of the overall freight demand network in six provinces, and they have the greatest impact on the freight transport of other cities in six provinces of central China. From the perspective of local distribution characteristics, there are two “chain reaction circles” in the freight demand network: the first is in the south of the six provinces in central China, with Wuhan, Hefei, and Nanchang as the center. The three cities jointly influence the freight demand network of Puyang, Changsha, Shangqiu, Zhoukou, Anqing, and other cities, to form a “chain reaction circle” in the form of a rhomboidal spatial structure (Fig 2a). The second one is located in the north of the six provinces, with Zhengzhou and Taiyuan as its center. The two cities jointly influence cities such as Luliang, Yuncheng, Yangquan, and Anyang, and its influence is smaller than the southern “chain reaction circle” (Fig 2a), as related to the weaker spread of the epidemic in the northern cities of six provinces in central China compared with the situation in southern China.

Fig 2a shows the first contact method of the city with the strongest connection strength with each city. From the perspective of the first demand contact network for freight, the “multi-center-radiation” pattern centered in Wuhan, Zhengzhou, Nanchang, Hefei, Taiyuan, Puyang, and Changsha is obvious. There are nine cities with Wuhan as the first connected city (Xiangtan, Hengyang, Yongzhou, Shaoyang, Loudi, Huaihua, Pingdingshan, Yichun, and Chuzhou), and five cities with Hefei as the first connected one (Yueyang, Ji’an, Puyang, Suizhou, and Anqing), accounting for 18% and 10% of all cities in six provinces of central China, respectively. The first contact area of other central cities is relatively small, indicating that the freight demands of nine cities including Xiangtan are most affected by the Wuhan central node. After the closure of the city, Wuhan became a choke-point that caused imbalance and even shocks in the freight market in nine cities including Xiangtan. Wuhan’s freight demand network was cut off and nine cities were involved, its freight market-related activities were severely affected. Hefei (from February 6 to February 18, 2020, the number of confirmed cases was 123) is the highest in Anhui Province. After the city’s “closure management” measure was adopted in Hefei, some trains were suspended, and traffic and roads were restricted. The result is that urban freight network was implicated, and five cities including Yueyang were affected by the chain effect of the partial cut-off of the central node in Hefei.

Through the calculation and analysis of the impact network of freight demand of each city, the preliminary conclusion can be drawn: Wuhan is the most influential city in the freight demand network, followed by Zhengzhou and Hefei, forming a multi-center structure in the freight demand influence network of six provinces in central China. Among them, the freight impact capacity of Wuhan is 11.6 times the average value of six provinces in central China, and 1.3 and 1.2 times that of the secondary centers of Hefei and Zhengzhou, respectively. This shows that after the closure of the city, Wuhan, as a comprehensive freight hub of six provinces in the central China, has cut off its traffic, leading to the imbalance on the freight demand network of the six provinces. Wuhan is the first choke-point for the imbalance and turbulence of freight demand network in six provinces in central China: Hefei and Zhengzhou are secondary choke-points.

## Analysis of a complex network of freight demand in six provinces of central China

### A complex-network topology structure

This study demonstrates the complex-network topology structure of freight demands of six provinces in central China from three dimensions: centrality-topological structure analysis, correlation analysis between network measurements, and core-periphery analysis.

#### The complex-network centrality topological structure

**Fig 3:**
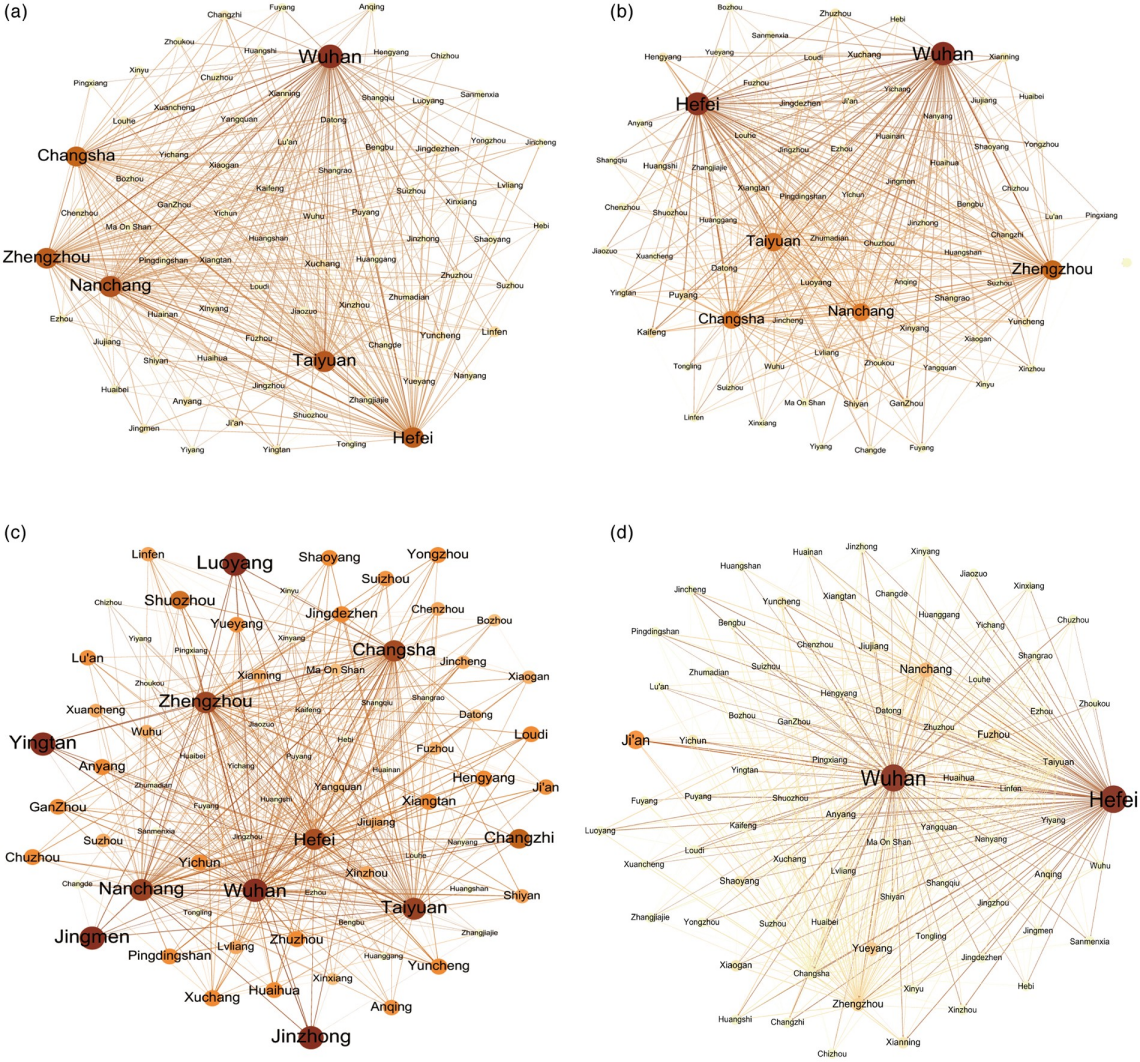
Complex-network topology structure of freight demand. a. Degree centrality. b. Weighted degree centrality. c. Closeness centrality. d. Betweenness centrality.

We use Gephi software for calculating the degree centrality, weighted degree centrality, closeness centrality, and betweenness centrality (Fig 3), to show the topological structure of the freight demand network of six provinces in central China.

#### Degree centrality

Degree centrality is the most direct measurement indicator of node centrality in complex network analysis. The larger the degree centrality, the higher the node centrality, and the more important the node is in the complex network. It represents the centrality and dominance of a city in the freight demand network. The larger the value is, the more critical the position of the city in the freight demand network. Degree centrality is further divided into two types: in-degree and out-degree. In-degree reflects the demand for goods input (*i*.*e*., “demand” in the balance between supply and demand); out-degree reflects the demand for goods output (*i*.*e*., “supply” in the balance between supply and demand). Based on the calculation of degree centrality, Fig 3a shows that the central nodes and values of the degree centrality network are Wuhan (96), Nanchang (80), Taiyuan (79), Zhengzhou (77), Hefei (74), and Changsha (71). Among them, Wuhan represents the first choke-point (Nanchang ranks second). The in-degree values of Wuhan, Nanchang, Taiyuan, Zhengzhou, Hefei, and Changsha exceed the out-degree values. The six provincial capitals form a pentagon and spread to affect other cities in the six provinces of central China. The primary factor of the imbalance of the freight demand market is that the output demand of goods is much greater than the input demand of small and medium freight enterprises in the six provinces of central China (the out-degree value on average is 9.07 which is greater than the average in-degree value of 1.62). This indicates that the backlog of goods for output became more serious during the COVID-19 pandemic.

**Fig 4:**
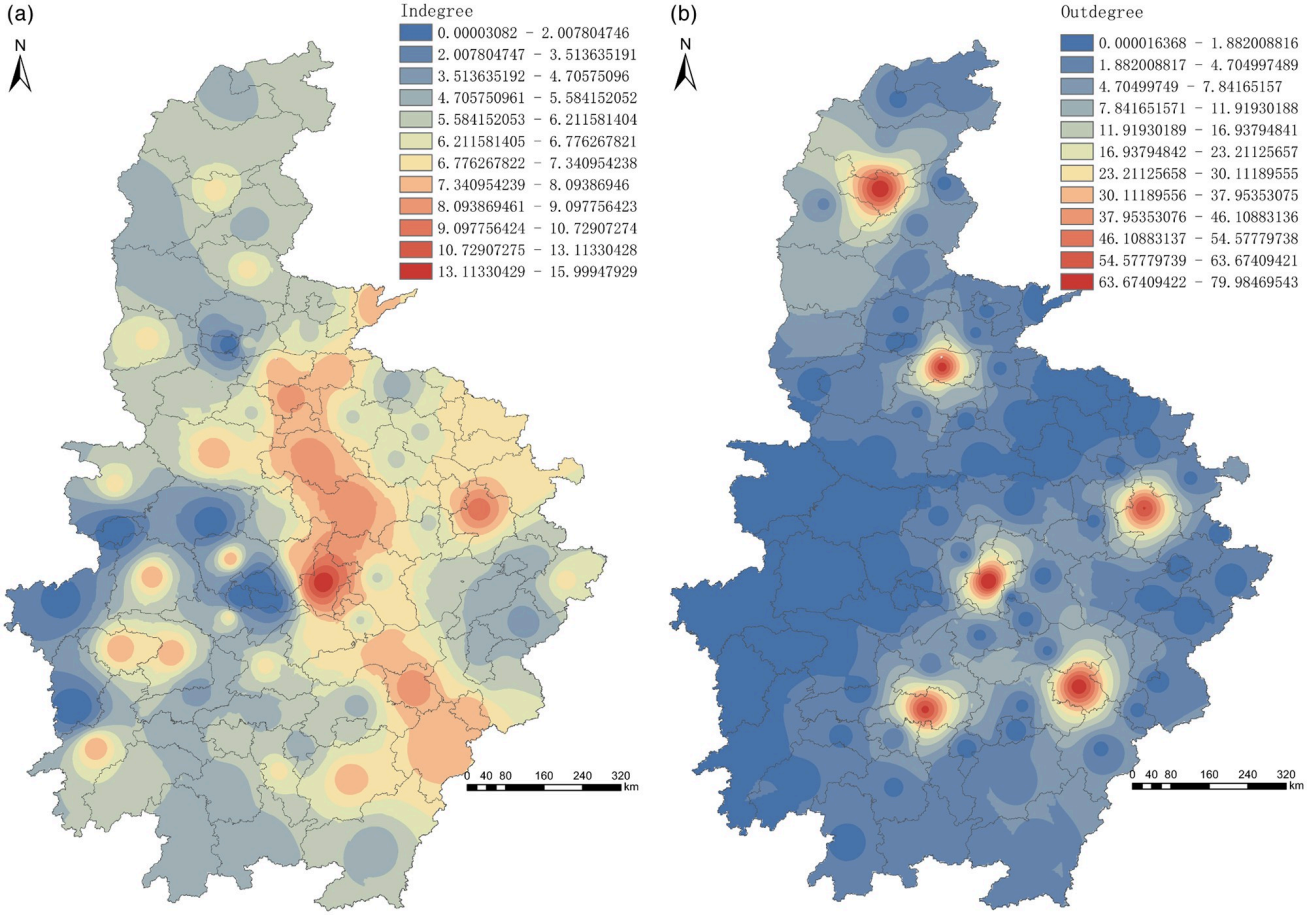
Spatial distribution of in-degree and out-degree in six provinces in central China. a. In-degree. b. Out-degree.

Fig 4 illustrates that the spatial heterogeneity of the cargo output network is very significant, presenting an island distribution. The out-degree values of Wuhan (79.98), Taiyuan (71.62), Nanchang (70.15), Zhengzhou (68.46), Changsha (65.84), and Hefei (63.71) are the highest. The demand for goods output is relatively strong. However, the spatial heterogeneity of the cargo input network is not obvious in the freight demand network of the six provinces in central China, and the difference of degree value between cities is small, which means they are uniformly distributed. The sum of in-degree values in the first six sites accounts for 11.78% of the total, while the out-degree values account for 78.21%. It discloses that the input freight demands of cities are scattered, and the demands for goods input remain too weak. During the spread of the disease, anti-epidemic materials and necessities in Wuhan and other cities are transported and supplied by the state. The small and medium freight enterprises (accounting for about 70% of China’s freight transport enterprises) suffer a serious imbalance between supply and demand, especially in terms of blocked demand for goods output.

**Fig 5:**
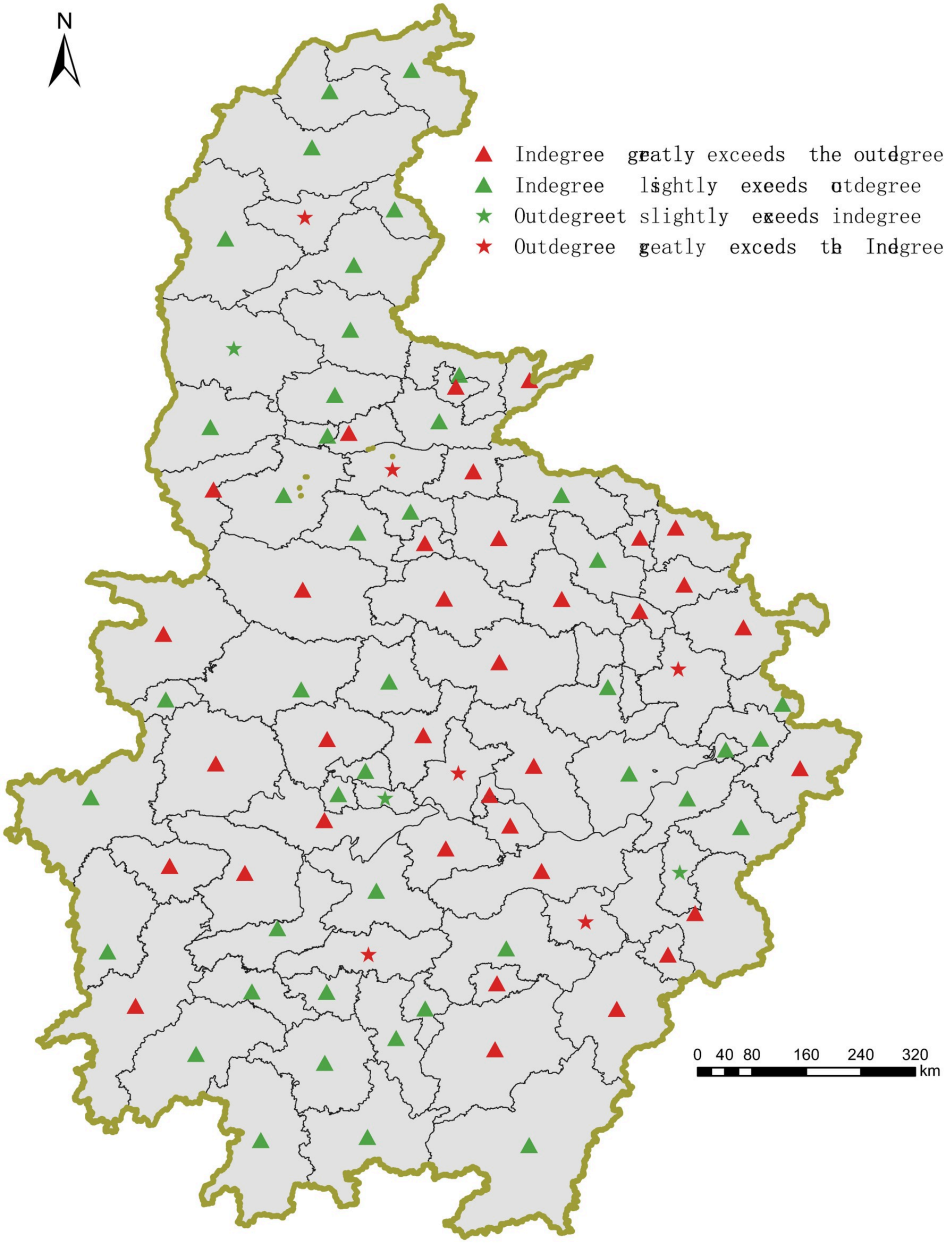
The differences in the spatial distribution of out-degree and in-degree values.

Fig 5 highlights that the out-degree values of 17 cities are greater than their in-degree values, of which six provincial capital cities have a larger difference, especially Taiyuan (the difference is 65). 66 cities’ out-degree values minus corresponding in-degree values return a negative value, such as for Xinyang, Zhumadian, Changde, Zhangjiajie, Jiujiang, Jingmen, Xiaogan, and Fuzhou, but the differences are minor, and the maximum difference value among them is -9 in Zhumadian and Xinyang. This indicates that another reason for the imbalance between supply and demand in the freight market lies in the large difference between freight input and output among different cities and the uneven distribution.

#### Weighted degree centrality

Weighted degree centrality considers the connection of weights. In the weighted centrality network, the higher the weight of a city, the higher the level of its centrality. Fig 3b shows that the cities with the highest weighted degree centrality are Wuhan (40,952.13) and Hefei (39,492.54), both of which are located at the highest level of the intercity freight demand network. The two cities are also choke-points in the imbalance of the freight demand market, which can determine the imbalance and development of the whole weighted degree centrality network. Zhengzhou (30,112.00) and Taiyuan (27,170.78) are the secondary choke-points of the imbalanced freight market.

#### Closeness centrality

Closeness centrality is used to describe the difficulty of inter-node transit through the network, representing the sum of shortcut distances between a point and all other points. If a journey from one node to other cities is easy, then the node can be considered as having good accessibility. High closeness centrality indicates that the city has a strong demand for freight accessibility and urgently needs to realize smooth transportation. Fig 3c shows that Wuhan, Taiyuan, Nanchang, Changsha, and Hefei have the most vigorous demand for freight accessibility, and the congestion values of their freight channels are also the highest, with their closeness centrality being 0.97, 0.91, 0.90, 0.86, and 0.84, respectively. This shows that the accessibility of freight transport demands in these five cities is high, and the urban freight backlog is severe.

The spatial distribution of closeness centrality presents high-value aggregation in Shanxi Province. Cities whose closeness centrality is above average are concentrated in the north, central, and south-east of the six provinces in central China, such as Taiyuan, Wuhan, Nanchang, Hefei, Changsha, Luoyang, Zhengzhou, *etc*. Compared with betweenness centrality, closeness centrality has a balanced spatial distribution, showing a dispersed distribution with Wuhan and Hefei as dual-cores (Fig 4c).

#### Betweenness centrality

Betweenness centrality is used to measure the degree to which a point is located in the middle of other point pairs in a complex network, as a tool to reflect the ability and status of a node as a medium for other nodes in the network. Betweenness centrality mainly represents the control capability on the freight network resources and plays a decisive role in the process of highway freight transit. Therefore, those cities with high betweenness centrality are mostly transit stations and have strong transit demand in the freight network. Fig 3d illustrates that Wuhan and Hefei have the strongest demand for freight transit, with the betweenness centrality of 1142.05 and 1139.27, and many goods need to be transferred from the two cities. Ji’an, located in Jiangxi, is closely following, with a betweenness centrality of 541, and strong demand for freight transit. Ji’an is the one of top 100 foreign trade cities in China, plus with convenient transportation, so the freight volume is high. Under the influence of COVID-19, many goods are being transferred from Ji’an due to the mild epidemic situation compared with other cities in Jiangxi.

Overall, the geographical distribution of betweenness centrality is unbalanced. Wuhan and Hefei have greater weight in the overall network, accounting for 24.4% and 24.3% of the total value of betweenness centrality of six provinces in central China. The betweenness centrality network of the six provinces appears a typical dual-core structure (Fig 3d).

### Correlation analysis of freight demand network measurement in six provinces of central China

The different network centrality of six provinces in central China is calculated by Gephi software and the correlation coefficient is deduced by the Pearson function in the SciPy module of Python to analyze the correlation between network measurements in the freight network. The positive correlation is remarkable and the correlation coefficients between degree centrality and out-degree, weighted out-degree and weighted degree centrality, weighted degree centrality and betweenness centrality, as well as degree centrality and betweenness centrality are 0.99, 0.99, 0.69, and 0.58 respectively.

Out-degree values are greater than in-degree values in the degree centrality network, so the out-degree determines the development trend of the degree centrality network and output freight demand has a greater influence on the freight imbalance. In the weighted degree centrality network, weighted out-degree has a greater influence on the freight market. This means that Wuhan and Hefei, with higher hierarchy in the network, exert a diffuse impact on other cities in the six provinces in central China, and the related economic activities of freight enterprises in Wuhan and Hefei are affected, which is likely to trigger a chain reaction in the freight demand market of other cities at different levels. Wuhan and Hefei are the cities with the highest value of weighted degree centrality and betweenness centrality. This indicates that cities with high levels often take responsibility for goods transit in the freight demand network. This is to say, the cutting-off of the choke-point of the freight demand network will lead to the breakdown of the whole freight network. By comparison, the correlation between closeness centrality and betweenness centrality is weak and so does betweenness centrality and weighted centrality. This shows that there is not a strong correlation between freight accessibility and transit capacity. In other words, if the freight accessibility of a city is blocked, the smooth transportation of goods can be realized through transit via other cities. However, a city with a high degree of centrality and strong traffic radiation capacity does not mean that it has a high hierarchy in the freight demand network. For example, Wuhan, Changsha, Hefei, Nanchang, Zhengzhou, and Taiyuan have strong radiation capacity (Fig 3a), but only Wuhan and Hefei occupy the first level of freight demand network. Hence, the other four provincial capital cities should take measures to cope with the imbalance of the freight market, to prevent a series of chain reactions triggered by Wuhan and Hefei.

### Core-periphery analysis of the freight demand network

**Fig 6:**
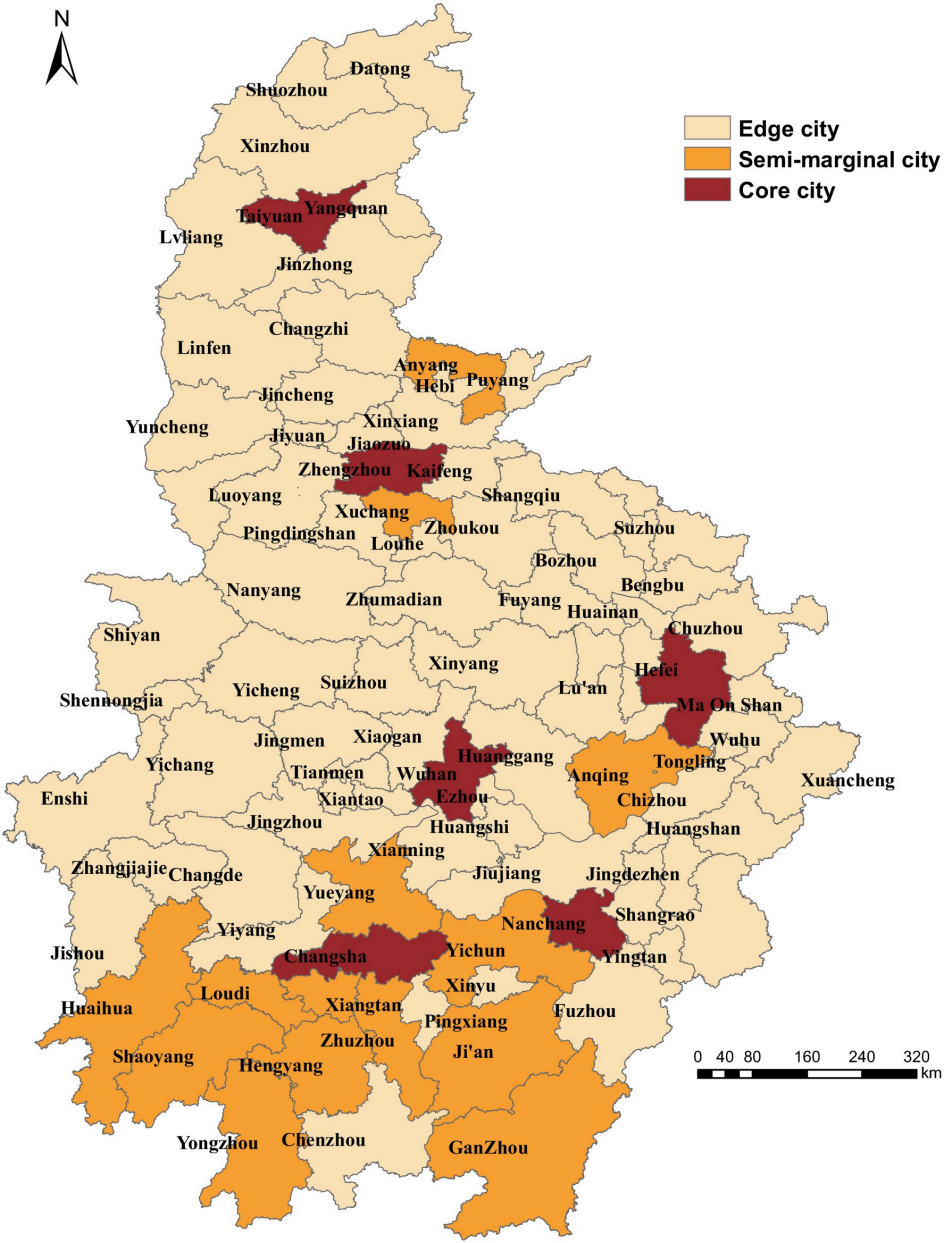
Core-periphery analysis of the freight demand network.

Cities are divided into the core, semi-periphery and periphery cities of the freight demand network using a continuous core-periphery model in UCINET software. The fitting value of the model is 0.933, with high confidence. According to the continuous core-periphery model, there are six core cities, namely, the six provincial capitals: Wuhan, Hefei, Zhengzhou, Changsha, Taiyuan, and Nanchang and 14 semi-peripheral cities showing a strong spatial adjacency effect in the south of the six provinces in the central region (Fig 6). The 63 periphery cities (such as Huangshan, Xuancheng, and Ma’anshan), are distributed in bands around the core cities, indicating that, once the six core cities have sustained more shocks, leading to the imbalance in freight transport, semi-peripheral cities are easily affected.

## Impact of epidemic evolution on the freight demand network of six provinces in central China

We demonstrate a correlation analysis between the number of COVID-19 confirmed cases of six provinces in central China from February 6 to 18, 2020, and network measurements including degree centrality, weighted degree centrality, closeness centrality, and betweenness centrality of the freight demand network.

The correlation coefficients between the number of COVID-19 confirmed cases and betweenness centrality, closeness centrality, degree centrality, and weighted degree centrality are 0.63, 0.22, 0.47, and 0.51, respectively. The correlation coefficient between betweenness centrality and the number of COVID-19 confirmed cases is the highest (0.63). On the one hand, this indicates that cases of COVID-19 will intensify due to multiple freight transit operations, on the other hand, it implies that the spread of COVID-19 may provide new opportunities for the improvement of transit function of small and medium cities. The correlation coefficient between degree centrality and the number of COVID-19 confirmed cases is 0.47. On the one hand, this means that the more serious the COVID-19 caseload in a city, the stronger the demand for goods, which is related to banned traffic and freight transportation missing after the lockdown of the cities. On the other hand, the cities with milder epidemic caseloads will have opportunities after Wuhan, Hefei, and other freight choke-points are cut off. The correlation coefficients between the number of COVID-19 confirmed cases and in-degree and out-degree are 0.48 and 0.44, respectively. This suggests that both the output and input of goods will affect the COVID-19 situation, and the goods output has a greater influence on the epidemic.

The correlation coefficient between the number of confirmed cases and the difference between out-degree value and in-degree value is only 0.068, implying no absolute correlation between the freight market imbalance and the epidemic. Perhaps the imbalance between supply and demand in the freight market of the six provinces in the central region has always existed, but the COVID-19 pandemic has struck the freight demand market, rendering it more complicated with a series of chain reactions.

### Imbalance and breakout: The imbalance under the spread of the epidemic

#### (1) Imbalance difference: The intensity of freight demand is seriously unbalanced, leading to a significantly uneven spatial distribution between the output and input of goods

According to the above analysis, it can be found that the freight demands in six provinces of central China exhibit a typical long-tailed distribution, with a large distribution span, and the proportions of freight demand intensity among cities are seriously unbalanced (Fig 1). During the spread of the epidemic, the spatial distribution heterogeneity of cargo output network in six provinces of central China is very significant, showing typical “island” distribution characteristics. The spatial heterogeneity of the goods input network is not obvious, and the difference of degree value between cities is small, implying that they are uniformly distributed. Overall, the demand for goods export is much greater than that for their import.

Freight output and input, namely supply and demand, are the most basic demand points in a freight demand network. The main reason for the imbalance between supply and demand in the freight market is the large difference in freight input and output in different cities, and the distribution is uneven. Freight enterprises in various cities exhibit large differences in the import and export demands of goods, and the supply and demand are imbalanced. Wuhan, Nanchang, Taiyuan, Zhengzhou, and Hefei, which are in the top rankings in terms of centrality, have far greater out-degree values than in-degree values. Those six cities form a pentagonal chain reaction area and spread the effect thereof to other cities in the six provinces of central China.

#### (2) Imbalance of chain reaction: There are multiple “chain reaction circles” in the state of weak freight connection

The inter-city freight links present an unbalanced network in a state of weak connectivity, affected by the closure of cities and roads during the pandemic. Although the connection is weak, Wuhan, Hefei, Zhengzhou, Nanchang, Zhengzhou, Taiyuan and Changsha, as the center, have formed two chain reaction circles in the north and south of the freight demand network. The influence of the northern “chain reaction circle” is smaller than that of the southern one, and the latter has spread widely, even affecting Shangqiu, Zhoukou, and other cities in the north. This finding shows that, even if the connection and radiation capacity of the seven freight hubs are weakened, the non-linear chain reaction, caused by epidemic shock, persists and propagates.

#### (3) The key to the imbalance: The choke-point is the crux of the imbalance and even shock of the freight demand network

Wuhan is the choke-point which leads to the imbalance and even turbulence in freight demand network, and it affects a wide range. Hefei and Zhengzhou are secondary choke-points; the other three provincial capitals have a profound influence. According to the analysis of the “core-periphery”, it indicates that once the six provincial capitals are disturbed, it will lead to the imbalance of freight transport, and the 14 semi-peripheral cities such as Pingdingshan and Anqing are easily affected. Therefore, the keys to preventing the chain reaction in freight demand networks are controlling the choke-point and secondary choke-points.

#### (4) Hidden danger of imbalance: The hidden danger of imbalance between supply and demand in freight market is gradually exposed

The difference between the out-degree and in-degree is not correlated with the number of confirmed cases. It shows that the imbalance between supply and demand in the freight market in six provinces of central China is not absolutely related to the epidemic. The hidden danger of the imbalance has existed for a long time and has been gradually exposed under the spread and shock of the epidemic. The epidemic is only the “fuse” on the imbalance in the freight market, and its violent shock has made the market more complex and intertwined and produced a series of chain reactions related to freight imbalance.

### Breakout in the post-epidemic era

#### (1) Freight transit breakout: Small and medium cities break through to form new freight transit stations

The correlation coefficient between betweenness centrality and the number of confirmed cases is the highest, which indicates that multiple transfers of goods will increase the spread of the epidemic. On the other hand, it shows that the spread of the epidemic may provide new opportunities for the promotion of the transit function in small and medium-sized cities. Ji’an, which ranks the third in terms of betweenness centrality, is close to Nanchang, the capital of Jiangxi Province and on the border of Jiangxi and Hunan. It bears traffic in all areas. With the spread of epidemic, the situation in Ji ’an, located in Jiangxi Province, is relatively mild. Enjoying a superior geographical position, Ji’an can improve its freight transit capacity and seek breakout and development in the post-epidemic era.

#### (2) Freight balance breakout: Eliminate hidden dangers, and actively promote the balance between import and export of goods in the six provinces of central China

It can be found that the primary factor of the imbalance in the freight demand market is that the demand of goods output is much greater than input (the average out-degree is 9.07 which is greater than the average in-degree of 1.62) implying that they are extremely unbalanced. “Ice a meter deep is not frozen in one day”, as the saying goes, the difference between the out-degree and in-degree is not correlated with the number of confirmed cases, and the hidden danger of the imbalance between supply and demand may endure. Therefore, to avoid the imbalance and even turbulence of the freight demand network, it is necessary to promote the balance of input and output in the freight market in the post-epidemic era, eliminate the hidden dangers and seek breakout and development opportunities.

#### (3) Freight intensity breakout: Take advantage of geographical proximity, tap freight potential, increase freight volume, and explore new economic growth points

From the perspective of the first demand contact network for freight, the “multi-center-radiation” pattern centered on Wuhan, Zhengzhou, Nanchang, Hefei, Taiyuan, Puyang, and Changsha is obvious. Puyang’s freight frequency ranks among the top cities in six provinces of central China, with strong goods input (freight frequency and in-degree are both ranked in the top ten). Puyang has an obvious geographical proximity advantage, as it is located at the junction of Henan, Hebei, and Shandong Provinces and sits adjacent to Liaocheng, Tai’an, Handan, Anyang, and Xinxiang and other cities, and Daguang Expressway, Puhe Expressway, Nanlin Expressway, and Pufan Expressway run through the whole city. The weakness of Puyang’s freight demand network lies in its small freight volume, while its advantage lies in its high freight frequency, so its freight potential needs to be further developed. In the post-epidemic era, relying on Puyang’s advantages in terms of highway accessibility and geographical proximity, its freight network can be expanded. By improving the accessibility of its local freight network, the development of freight enterprises in Puyang can be promoted.

Other cities akin to Puyang are Xuchang and Kaifeng: Xuchang is a transportation and logistics hub city in the Central Plains Economic Zone, bordering Zhengzhou in the north and Kaifeng in the east, with superior geographical position and developed transportation and logistics. Kaifeng, bordering Zhengzhou in the west, is one of the central cities in the core area of Central Plains urban agglomeration approved by the State Council. Kaifeng Free Trade Zone is one of the three major areas of China. Xuchang and Kaifeng are both adjacent to Zhengzhou, a comprehensive logistics hub. In the post-epidemic era, we can consider following the “Ningbo-Zhoushan” model to determine new economic growth points in Xuchang and Kaifeng and build a “Zhengzhou-Xuchang-Kaifeng” highway freight hub. Through industrial transfer, the surplus freight volume of Zhengzhou will be transferred to Xuchang and Kaifeng, and the freight absorption capacity of the two cities will be improved. Zhengzhou develops its freight development and improves the volume in transit, to promote the development of its small and medium freight enterprises. The formation and development of a new urban agglomeration centered on Zhengzhou can then be promoted.

#### (4) Breakout of freight in peripheral cities: Improve the freight input function and enhance the ability to receive more traffic

Based on the above analysis, during the epidemic, there are 17 cities with mainly export demand, especially provincial capitals. There are 66 cities that mainly import, but the demand is not high. The maximum levels occur in Zhumadian and Xinyang (at -9). This shows that there is a certain potential for the import of goods in the six provinces of Central China. The 66 cities with import demand as the main factor do not have a strong external freight influence but have a strong ability to receive traffic, so their importance in the freight network is relatively limited. In the post-epidemic era, these cities can consider expanding the freight market demand, and absorb from other major export cities. Cities that focus on import are suggested to guide freight companies to form a “freight alliance” with those in cities that focus on export demand to eliminate the hidden dangers of freight imbalance. Most of periphery cities dominated by import demand, and the development of small and medium freight companies in those cities have become more difficult since the outbreak. In the post-epidemic era, the “freight alliance” can be used to eliminate the hidden dangers of freight imbalance, allowing small and medium freight companies in peripheral cities to seek breakout and development.

## References

[1] Liu HM, Fang CL and Gao Q. Evaluating the Real-Time Impact of COVID-19 on Cities: China as a Case Study. Complexity. 2020. Available from 10.1155/2020/8855521.

[2] Wang Y. The epidemic will promote global supply chain reform. Chinese Social Sciences Today. 30th March,2020.

[3] Xu XL et al. An analysis of the domestic resumption of social production and life under the COVID-19 epidemic. Plos one. 2020. Available from 10.1371/journal.pone.0236387. 32697812 PMC7375597

[4] Arellana J, Márquez L and Cantillo V. COVID-19 Outbreak in Colombia: An Analysis of Its Impacts on Transport Systems. Journal of Advanced Transportation. Available from 10.1155/2020/8867316. 32908973

[5] J Jia J S, Lu X, Yuan Y, et al. Population flow drives spatio-temporal distribution of COVID-19 in China[J]. Nature, 2020, 582(7812):1–11. Available from 10.1038/s41586-020-2284-y. 32349120

[6] Yang P and Shi F. Urban logistics storage facilities equilibrium model based on commodity supply and demand network and is application. Systems Engineering-Theory&Practice,2012, 32(8): 1681–1691.

[7] Liu X, Chen G, Sheng Z, Analysis on food safety and government regulation under the different relationship between supply and demand[J]. Chinese Journal of Management Science, 2010,18(2):143–150.

[8] Zhang Q, Dou Y, Chen S. The combined matching model of road freight supply and demand information[J]. Statistics & Decision, 2018, 34(14):72–75.

[9] Yamada T, Imai K, Nakamura T, Eiichi T. A supply chain-transport supernetwork equilibrium model with the behaviour of freight carriers[J]. Transportation Research Part E Logistics & Transportation Review, 2011, 47(6):887–907.

[10] Cao X, Li T. Evolution of spatial pattern of integrated Transport efficiency in urban agglomerations [M]. Beijing: The Commercial Press.2017.

[11] Li L, Ma X. Pattern, stricture and function of China’s express logistics network based on waybill data:a case study of ZJS Express[J]. Scientia Geographica Sinica, 2019, 39(1):92–100.

[12] Mi Z, Zeng G. Logistics Linkage Pattern, characteristics and influencing factors of the Yangtze River economic belt under different scales[J]. Scientia Geographica Sinica, 2018, 38(7):1079–1088.

[13] Yang J and Yao C. Research on logistics industry competitive relationship model based on complex networks[J]. Chinese Journal of Management, 2010, 7(3): 406–411.

[14] Zhang Y. The application of the complex network theory in logistics network[J]. China Business and Market, 2011, 25(5):38–42.

[15] Zhang X. Miller-Hooks E, Denny K. Assessing the role of network topology in transportation network resilience[J]. Journal of Transport Geography, 2015, 46:35–45.

[16] Dubie M. et al. An evaluation of logistics sprawl in Chicago and Phoenix. Journal of Transport Geography. 2018. Article in Press. 10.1016/j.jtrangeo.2018.08.008.

